# Immunotherapy – 2069. Multi-center study on the performance characteristics of two skin test devices - Comforten® and Mult-Test II®

**DOI:** 10.1186/1939-4551-6-S1-P152

**Published:** 2013-04-23

**Authors:** Rohit K Katial, Linda Cox, Derek Constable

**Affiliations:** 1 Allergy & Immunology, National Jewish Health; 2 Allergy & Asthma Center, Ft, Lauderdale, FL, USA

## Background

Two multiple test devices, ComforTen (CF10-HollisterStier Allergy) and Multi-Test II (MT-Lincoln Diagnostics), were compared at two allergy clinics. The information generated may assist clinicians in making an informed decision when selecting a skin testing device.

## Methods

Subjects at each site (24-AAC/16-NJH) were blind skin-tested on the back with each device in duplicate using a negative control and two histamine positive controls(1 and 6mg/mL). Wheal sizes were recorded (mm) after 10 minutes. After each test, subjects were asked to rate the pain and on two occasions test preference.

## Results

Overall, CF10 gave smaller wheals than MT, (combined sites and both histamines1.95 vs. 3.53, p<0.001). Wheals were also smaller at NJH than at AAC (combined devices and histamines 2.33 vs. 3.16, p<0.001). Comparing device- histamine combinations as described by each company’s product insert, i.e. CF10 with 6mg/mL vs. MT with 1mg/mL, wheals were not significantly different (combined centers 4.00 vs. 4.07, p=0.62). The impact of wheal size on sensitivity and specificity to define a positive reaction was examined at 1, 3 and 5mm. Sensitivity increased as the cut-off decreased and trended higher for MT than CF10. Specificity was high (100%) for all cut-off levels at NJH but lower at AAC for MT (80%, 85% and 92%) and CF10 (98%, 98% and 99%). Optimal performance across sites showed that both devices required 6mg/mL histamine but with device specific cut-offs CF10-1mm (sensitivity=93%, specificity=99%), and MT-3mm (sensitivity=94%, specificity=91%). Pain using the two devices appeared to be site-specific. At ACC, there was significantly lower pain using CF10 than there was using MT (0.77 vs. 1.68, p=0.001) while at NJH, there was no significant difference in pain scores. Overall, 57% of subjects showed preference to using CF10, while only 9% preferred MT. The remainder (34%) showed mixed preference.

## Conclusions

Both devices produced similar average wheal sizes when used as instructed by the manufacturer with their stated histamine concentration. However optimal results show device specificcut-off criteria usingthe 6mg/mL histamine control. Differences in operator techniques may account for the observation of some inter-site differences which highlights the importance of training.Studies were funded by grants from Jubilant HollisterStier LLC.

